# Insights into ectodomain shedding as a novel target in natural killer cell-based immunotherapy for cancer

**DOI:** 10.3389/fimmu.2026.1753470

**Published:** 2026-03-09

**Authors:** Ruan Pimenta, Jun Arai, Luiza Ribeiro de Lima Brandao, Pascale Schmidt, Bruna Taciane da Silva Bortoleti, Lucas Ferrari de Andrade

**Affiliations:** 1 The Marc and Jennifer Lipschultz Precision Immunology Institute, Icahn School of Medicine at Mount Sinai, New York, NY, United States; 2 Department of Gastroenterology, Aichi Medical University, Nagakute, Japan; 3 Department of Immunology and Immunotherapy, Icahn School of Medicine at Mount Sinai, New York, NY, United States; 4 Department of Oncological Sciences, Icahn School of Medicine at Mount Sinai, New York, NY, United States; 5 The Tisch Cancer Center, Icahn School of Medicine at Mount Sinai, New York, NY, United States

**Keywords:** altered self, antibody-dependent cellular cytotoxicity, cancer immunology, cancer immunotherapy, ectodomain shedding, natural killer cells, proteolytic cleavage

## Abstract

Natural killer (NK) cells are innate lymphocytes that kill cancer cells and produce cytokines/chemokines that drive anti-tumor immune responses. NK cells are controlled by activating and inhibitory receptors, of which some, or their ligands, are regulated by ectodomain shedding, which is a post-translational modification to transform surface proteins into soluble peptides. Although ectodomain shedding is essential for normal development and physiology, it is also an immune suppression mechanism for downregulating activating receptors in NK cells or their ligands in cancer cells. The ectodomain shedding of immune receptors or ligands are therapeutic targets in cancer immunology and peculiarly relevant in NK cells, given how receptor/ligand density tips the balance to the activation or inhibition of NK cell effector functions. Two classical examples are the CD16a Fc gamma-activating receptor and the MICA/B, and B7-H6 cellular stress-induced ligands. CD16a triggers antibody-dependent cellular cytotoxicity (ADCC), but CD16a shedding by ADAM17 can prevent receptor engagement, and, therefore, CD16a shedding is a target to promote the efficacy of Fc-enabled antibodies. CD16a shedding also appears to play a dual role, not only in negatively regulating ADCC but also in terminating the immune synapse to help NK cells disengage and move to the next target cell. Furthermore, stress-induced ligands serve as “kill me” signs on the surface of cancer cells, but the shedding of such ligands enables escape from NK cell recognition. Although the shedding of stress-induced ligands is a mechanism of immune evasion in tumors, novel monoclonal antibodies that inhibit such shedding in a highly specific manner have an outstanding efficacy in preclinical tumor models, and one clone has transitioned to clinical trial phase for potently promoting anti-tumor immunity. We, therefore, review here some of the most impactful discoveries in the ectodomain shedding field with a special focus on NK cells and cancer to help inform the scientific community and help guide the development of novel immunotherapies.

## Introduction

Ectodomain shedding is a post-translational modification whereby surface proteins undergo proteolytic cleavage in the stalk region by metalloproteases, which, as a consequence, release the entire extracellular domain (1). Ectodomain shedding transforms surface proteins into soluble peptides or polypeptides. Although ectodomain shedding is essential for normal development and homeostasis, it is also a mechanism of immune regulation that interferes with anti-tumor immunity (2). One key example is the natural killer (NK) cell, which has its effector functions regulated by a balance of activating and inhibitory stimuli; the ectodomain shedding of ligands for activating receptors decreases the number of engagement events, and, as a consequence, it interferes with the triggering of NK cell effector functions (3). Furthermore, recent developments have revealed that NK cells are promising therapeutic targets for cancer, such as through novel drugs that directly or indirectly stimulate NK cell-driven tumor immunity and chimeric antigen receptor NK cells (4). There are more than forty clinical trials investigating NK cell-based therapies for cancer (4). On the other hand, the aggregate of scientific literature indicates that ectodomain shedding is a novel target to therapeutically promote NK cell-driven immunity against cancers. We, therefore, provide a brief review of some of the latest and/or most impactful discoveries in the shedding of NK cell-related ligands and receptors, to help inform basic scientists in cancer immunology and guide the development of novel immunotherapies for cancer.

## Immune evasion through the shedding of stress-induced ligands

Cellular stress pathways can induce the expression of surface proteins that serve as ligands for NK cell-activating receptors, which trigger the effector functions (5). This mechanism of immunity is known as “altered self” and enables an NK cell-mediated clearance of virus-infected or malignant cells, which are often under endoplasmic reticulum, oxidative, and/or replication stress (6–8). However, many of the stress-induced ligands can undergo ectodomain shedding by metalloproteases that, for downregulating the expression of surface stress-induced ligands, enable an escape from NK cell recognition (9, 10). Furthermore, the shedding generates soluble stress-induced ligands that may have biological activity, such as by serving as decoys (11, 12). NK cell intrinsic dysfunctions, such as the one characterized by downregulation of activating receptors and effector molecules (*e.g.*, Fc receptor and granzyme B, respectively) in triple-negative breast cancer, can also contribute to immune evasion, thus highlighting the existence of extrinsic mechanisms (*e.g.*, stress-induced ligand shedding) and intrinsic mechanisms (*e.g.*, NK cell dysfunction) that may function coordinately to promote immunosupression (13). Therefore, cellular stress pathways induce immunity against abnormal cells, but the shedding of stress-induced ligands causes immune escape. We, here, provide a description of how ectodomain shedding interferes with two major pathways for NK cell-mediated surveillance against the altered self.

## Proteolytic shedding of ligands for the NK cell group 2 member D receptor

NKG2D is an activating receptor that induces NK cell effector functions and co-stimulates T cells (14–17). Its ligands in humans are major histocompatibility complex class I polypeptide-related sequences A and B (MICA/B) and the six members of the UL16-binding protein (ULBP) family, and all of those NKG2D ligands are expressed in response to cellular stress pathways. On the other hand, NKG2D ligands are rarely expressed in healthy tissues (7). NKG2D and its ligands, therefore, represent a mechanism for cancer immunosurveillance. However, the surface expression of some NKG2D ligands, *i.e.*, MICA/B and ULBP2, can be downregulated by ectodomain shedding (11, 18). Although the shedding of ULBP2 appears to occur upon NK cell-mediated tumor cell death, MICA/B shedding is a well-recognized mechanism for cancer immunoevasion and immunosupression (19, 20). For example, MICA/B shedding downregulates surface MICA/B and thereby prevents NKG2D recognition. Furthermore, MICA/B shedding generates soluble MICA/B molecules that bind NKG2D and cause NK cell desensitization, likely through chronic stimulation or exhaustion (11, 21–23). Serum or plasma MICA/B molecules are well-recognized biomarkers of cancer progression and immunotherapy response (20). Furthermore, tissue characterization analysis can complement serum biomarkers, to more comprehensively define the clinical importance of ectodomain shedding. In prostate cancer (PCa), for example, there is a progressive decrease in MICA/B expression on tumor epithelial cells with increasing histological grade, and that is accompanied by elevated serum MICA/B concentration in advanced disease (21). Furthermore, low-grade tumors can be characterized by MICA/B surface expression, but with progression to high-grade and castration-resistant phenotypes, the MICA/B expression pattern changes, with a redistribution to the stroma and concomitant loss of MICA/B surface expression (24, 25). Patients with advanced PCa have more soluble MICA/B in the plasma than patients with localized disease, and elevated levels of serum MICA/B correlated with NK cell dysfunction and metastatic disease (21, 24, 26, 27). Therefore, MICA/B shedding, for causing immunoevasion and immunosuppression, is a therapeutic target in cancer immunology research.

The MICA/B proteins are shed by the combined action of a disulfide isomerase (*i.e.*, endoplasmic reticulum protein 5, ERp5) and metalloproteases, such as a disintegrin and metalloprotease (ADAM) 10 and ADAM17 (10, 28–31). Furthermore, the MICA/B proteins are shed by membrane type matrix metalloprotease (MMP) 2 in renal cell carcinoma cells, MMP9 in osteosarcoma cells, and MMP14 in prostate and breast cancer cells (32–34). ERp5 interacts with the alpha-3 domain of MICA/B and appears to cause a conformational change that enables protease-mediated cleavage in the stalk, which is the linear peptide sequence in between the alpha-3 and transmembrane domains (10). On the other hand, protease inhibitors can stop MICA/B shedding, such as the anti-fungal drug lomofungin that can serve as an ADAM17 inhibitor to stop MICA/B shedding by liver cancer cells (35). However, a limitation is that proteases, like MMPs and ADAMs, have multi-substrate specificity (36). Therefore, protease inhibitors may cause pleiotropic effects if administered *in vivo* due to an inhibition of shedding of unintended targets beyond MICA/B.

**Figure 1:**
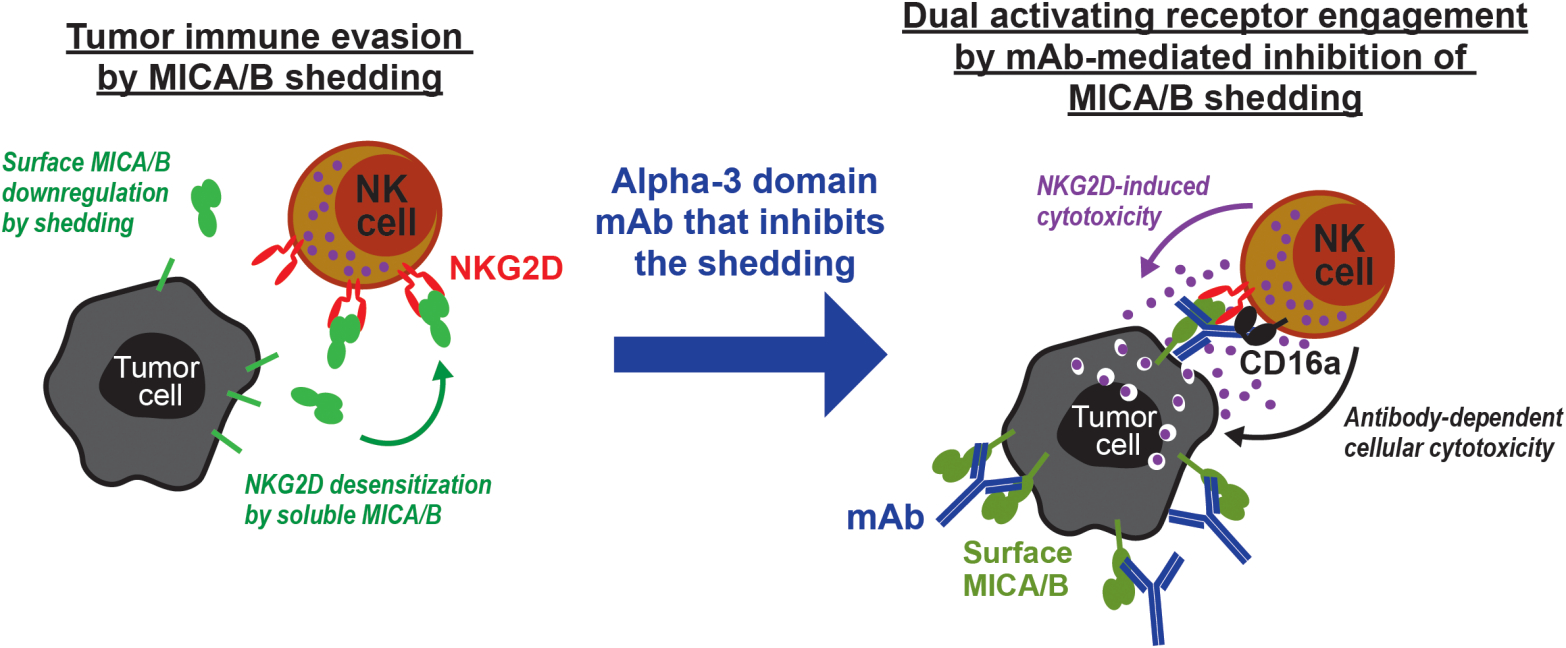
MICA/B shedding causes tumor immune evasion, but mAb-mediated inhibition of MICA/B shedding promotes NK cell-driven tumor immunity. NK cell-mediated cytotoxicity requires that abnormal or target cells are first recognized by NK cells through surface ligand and receptor interactions. The NKG2D-MICA/B immunosurveillance mechanism is a key example. NKG2D is an activating receptor that promotes NK cell effector functions against MICA/B-expressing cancer cells. However, cancer cells can proteolytically shed surface MICA/B, which is then downregulated for an escape from NKG2D recognition. Soluble MICA/B molecules were also shown to bind NKG2D and serve as a decoy. On the other hand, MICA/B shedding can be inhibited by protease inhibitors or, as illustrated here, certain alpha-3 domain-targeted mAbs that retain MICA/B on the cell surface for NKG2D recognition. If constructed with an Fc, such as human IgG1, which binds CD16a with relatively higher affinity, these mAbs can also trigger ADCC. Such mAbs can also engage Fc receptors in other leukocytes, such as macrophages that perform antibody-dependent cellular phagocytosis (not illustrated here). Therefore, antibody-mediated inhibition of MICA/B shedding is a pharmacologically relevant means to promote anti-tumor immunity.

Shedding-resistant MICA/B could be genetically engineered and expressed in cell lines by the introduction of six-point mutations in the alpha-3 domain for the proof-of-concept that the alpha-3 domain is a target to inhibit the shedding (37). Furthermore, alpha-3 domain-targeting monoclonal antibodies (mAbs) were developed specifically to inhibit MICA/B shedding. Such mAbs, for inhibiting the shedding, retain MICA/B on the tumor cell surface and, as a consequence, promote NKG2D recognition. 7C6 is one of them and a well-characterized clone. If engineered with enabled Fc, such as human IgG1 and mouse IgG2a, 7C6 not only promotes NKG2D recognition of tumor cells but also induces antibody-dependent cellular cytotoxicity (ADCC) (**Figure 1**) (38). Even tumors that were genetically engineered to escape CD8 T cell recognition were effectively treated by such an NK cell-mediated mechanism that is induced by 7C6 (39). Fc-enabled 7C6 also engages Fc gamma-activating receptors in myeloid cells, such as macrophages that perform antibody-dependent cellular phagocytosis (ADCP), and inhibits the leukemia outgrowth in acute myeloid leukemia (AML) models (40). Consistent with those promising preclinical studies, a novel MICA/B antibody that inhibits the shedding, *i.e.*, CLN-619, has transitioned to the clinical trial phase in patients with advanced solid tumors or multiple myeloma (NCT05117476 and NCT06381141) (41). In addition to that, the 7C6 sequence was developed as a chimeric antigen receptor (CAR) for cellular therapy (NCT07216105 and NCT07221032) (42). Therefore, antibody-mediated inhibition of MICA/B shedding, which can be achieved through certain alpha-3 domain-targeting mAbs such as 7C6 and CLN-619, represent novel and promising opportunities for cancer immunotherapy.

MICA/B shedding can also be therapeutically targeted through alpha-3 domain-based vaccines that elicit both NK and T cell-mediated immunity against cancers in preclinical models (43). An mRNA vaccine, *i.e.*, mCM10-L, which encodes a linear epitope-based peptide of the alpha-3 domain and a carrier protein, induces the generation of antibodies that inhibit MICA/B shedding and promote dual NK and CD8 T cell-driven tumor immunity against cancer (44). Furthermore, NKG2D ligand expression can be increased by chemotherapy or radiotherapy, which activate the DNA damage response and promote MICA/B mRNA expression in glioblastoma cells (45). It is, therefore, possible that the inhibition of MICA/B shedding, such as by the mAb 7C6, could be rationally combined with chemotherapy or radiotherapy that increase MICA/B mRNA expression to promote NK cell-driven immunity. In addition to inhibiting the MICA/B shedding, another therapeutic strategy to promote NKG2D-driven immunity consists of blocking soluble MICA/B through a neutralizing mAb that prevents NK cell desensitization (26, 46, 47). Therefore, recent developments in basic and applied research have made MICA/B shedding a pharmacologically targetable immunoevasion/immunosuppression mechanism for cancer immunotherapy.

NKG2D ligands can be shed not only through proteolytic cleavage but also in exosomes, which strongly downregulate NKG2D expression in NK cells (25, 48). Furthermore, MICA is shed not only in exosomes but also in medium size extracellular vesicles that, following prolonged stimulation, desensitize NK cells (49). Extracellular vesicle-mediated NKG2D ligand shedding has been reviewed elsewhere (50–52). Furthermore, NKG2D is expressed by T cells and provides co-stimulation (15, 16). MICB shedding suppressed the activation of NKG2D^low^ T cells in a pancreatic tumor model (53).

## Shedding B7 homolog 6 (B7-H6), a ligand for a natural cytotoxicity receptor

B7-H6, also known as NCR3 ligand 1 (NCR3LG1), is a type I transmembrane protein within the B7 family and functions as a ligand for the NK cell p30-related protein (NKp30), which is also known as NCR3 and serves as an activating receptor (54, 55). Consistent with the notion that B7-H6 is a stress-induced ligand, B7-H6 expression is rarely seen in healthy tissues, but it is upregulated in malignant or inflamed cells in response to cellular stress (8, 56). The NKp30-mediated recognition of B7-H6-expressing tumor cells triggers NK cell effector functions *in vitro* (54, 55). However, tumor cells can escape NK cell recognition by shedding B7-H6 through proteolytic cleavage (9, 57). In opposite contrast to the shedding of MICA/B, which is relatively well described, the molecular mechanisms underlying B7-H6 shedding are largely unknown, but they involve ADAM10 and ADAM17, and B7-H6 is only minimally shed in extracellular vesicles (9, 57). Soluble B7-H6 can be detected in plasma or serum samples, and it is increased in patients with, for example, advanced melanoma (9). Similar to how MICA/B shedding suppresses NKG2D, B7-H6 shedding also causes surface downregulation of B7-H6 that, in turn, enables immunoescape and generates soluble B7-H6 that binds and blocks NKp30 (9, 56). One key example is in ovarian cancer, where NK cells downregulate NKp30 likely through the chronic engagement with soluble B7-H6 in the peritoneal fluid (58). On the other hand, protease inhibitors stopped B7-H6 shedding and increased surface B7-H6 expression levels, which promoted NK cell-mediated killing of cancer cells *in vitro* but through the combined action of NKp30 and NKG2D, because NKG2D ligands are also shed by proteases (9). Therefore, B7-H6 is a “kill me signal” on the surface of tumor cells, but they escape NK cell recognition by shedding it through proteolytic cleavage, which not only downregulates B7-H6 surface expression but also generates soluble B7-H6 that is immunosuppressive for binding to NKp30 and desensitizing NK cells.

B7-H6 expression was associated with inflammation and carcinogenesis in chronic liver diseases, especially in metabolic dysfunction-associated steatotic liver disease (MASLD) and hepatocellular carcinoma (HCC). For example, patients with advanced MASLD, which includes patients with liver fibrosis and irreversible liver changes, have a significant increase in the concentrations of soluble B7-H6 molecules in serum (59). Soluble B7-H6 levels were also strongly correlated with biomarkers for HCC, indicating their potential for hepatocarcinogenesis (59). On the one hand, higher expression of B7-H6 in HCC tissues correlates with better post-operative prognosis (60). Furthermore, compared to non-neoplastic tissues in cirrhotic livers, B7-H6 expression was reduced in tumor tissues, and the concentrations of soluble B7-H6 in the serum were increased in HCC patients (61). Therefore, B7-H6 expression and shedding are peculiarly associated with liver metabolic disease and cancer.

## Dual contrasting roles of Fc gamma-activating receptor shedding

Antibody-dependent cellular cytotoxicity (ADCC) is a powerful mechanism of immunity whereby NK cells kill antibody-opsonized cells, such as tumor cells coated with therapeutic antibodies against tumor-associated antigens. ADCC is triggered by CD16a, which is an Fc gamma-activating receptor on the surface of NK cells (62). However, CD16a undergoes ectodomain shedding by ADAM17, which sheds it from the NK cell surface (63, 64). Because ADAM17 decreases the amount of CD16a molecules available for engagement with the antibody Fc, CD16a shedding is a well-recognized mechanism for immune suppression (63, 65–72). On the other hand, recent developments have also challenged such a paradigm by revealing that CD16a cleavage helps disassemble the immune synapse upon cytotoxicity and allows NK cells to disengage after degranulation (73, 74). Therefore, CD16a shedding appears to play a dual role in ADCC. We, here, describe many of the aspects of CD16a shedding-mediated regulation of ADCC and how it can be targeted for cancer immunotherapy.

## How CD16a shedding inhibit ADCC

To the best of our knowledge, the formal identification of soluble CD16a and CD16b in the human serum, by an enzyme-linked immunosorbent assay, dates back to the year of 1987 (75). For clarification, CD16b is an Fc gamma receptor expressed and shed by neutrophils, and with amino acid identity in the extracellular region that is nearly identical to that for CD16a, and thereby it is common that anti-CD16a mAbs cross-react with CD16b and vice versa (62, 76). Then, in 1989, Lanier et al. reported that human NK cells shed CD16a from the cellular surface and, as reported in 1991, through the action of metalloproteases (77, 78). Those initial breakthroughs, after approximately eleven years, were followed by the identification of ADAM17 as the protease that sheds CD16a from the NK cell surface (63). NK cells express ADAM17 and thereby the cleavage is likely in *cis* and it occurs in response to stimulations with certain cytokine or protein kinase C agonist treatments, as well as upon co-culture with tumor cells (63). The matrix metalloprotease 25 was also shown to cleave CD16a (79). The cleavage site or sites, as there may be more than one, of CD16a is (or are) in the stalk, which is a linear peptide sequence in between the membrane-proximal extracellular domain and transmembrane domain (66, 80). As a consequence of cleavage, NK cells lose membrane-anchored CD16a and shed soluble CD16a, which can be detected in the human blood circulation (81). On the other hand, protease inhibitors stop CD16a shedding and retain CD16a on the NK cell surface. As a consequence of protease inhibitor treatment, more CD16a molecules can engage the Fc of opsonizing antibodies that trigger ADCC (63). Furthermore, a mutant version of CD16a and that is resistant to cleavage, *i.e.*, CD16a-S197P, can be introduced into induced pluripotent stem cell (iPSC)-derived NK cells and that further confirmed that CD16a shedding inhibits ADCC, because the engineered NK cells had a better ability to kill tumor cells that were opsonized with Fc-enabled antibodies (66, 68). Such an approach has therapeutic potential for cancer and successfully transitioned to the clinical trial phase, for example, by incorporating the non-cleavable CD16a into a cellular therapy platform, such as the one with the 7C6 CAR (NCT07216105 and NCT07221032). Therefore, CD16a shedding, through the action of ADAM17 and upon NK cell stimulation, can inhibit ADCC by preventing receptor engagement.

**Figure 2:**
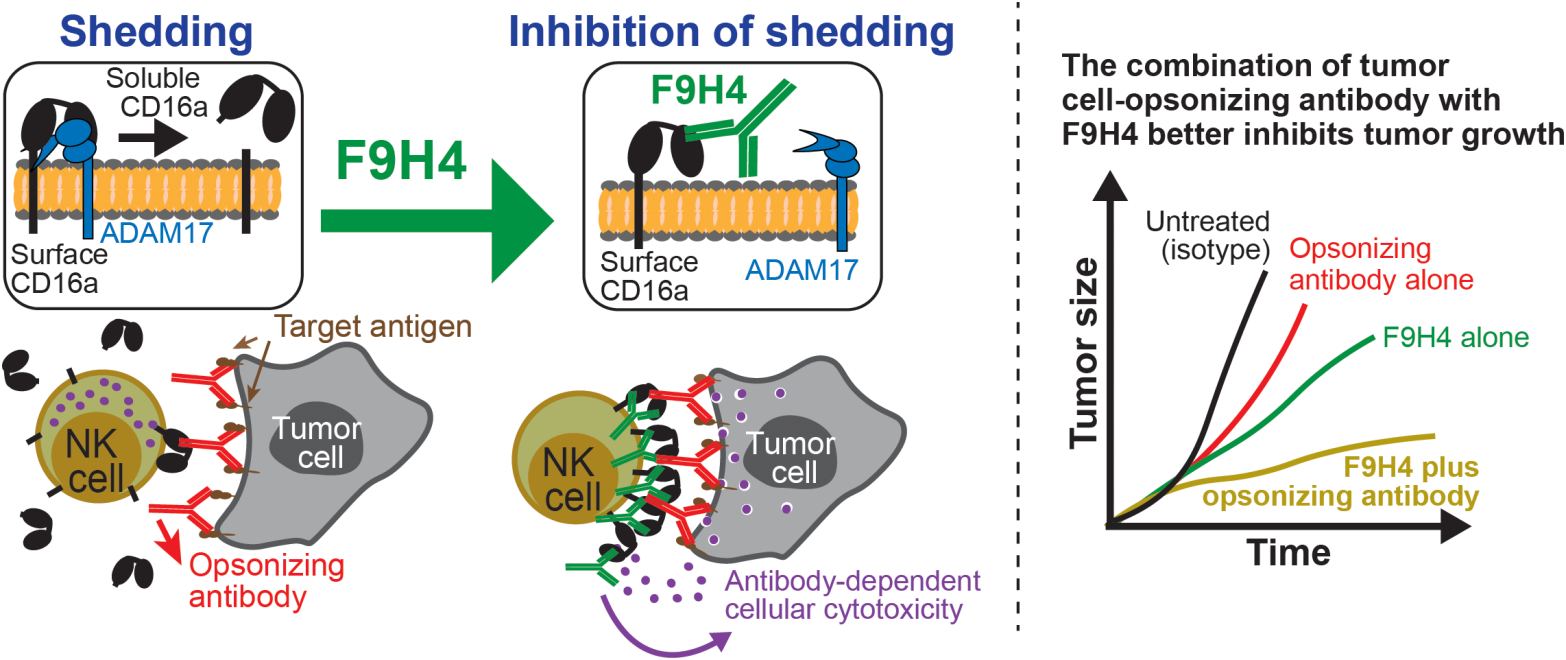
Surface CD16a can be downregulated by ectodomain shedding, but a novel mAb (F9H4) was developed to retain it for enabling engagement with the Fc. ADAM17 is the protease that cleaves the stalk region of surface CD16a, causing the shedding of most of the extracellular domain. As a consequence of CD16a shedding, fewer surface CD16a molecules are available in NK cells for detecting the Fc of tumor cell-opsonizing antibodies. On the other hand, the mAb F9H4 binds CD16a in a region that prevents blockade but inhibits the shedding, thus retaining CD16a on the NK cell surface for engagement with the Fc. As a consequence of this unique property, F9H4 promotes ADCC, which is a mechanism of immunity physiologically relevant for the immune control of tumor growth. In tumor models, F9H4 synergized with an Fc-enabled antibody that opsonized tumor cells, *i.e.*, cetuximab. In immunocompetent mice, F9H4 alone also inhibited tumor growth, likely by enhancing the ability of endogenous antibodies to promote ADCC.

Several therapies for cancers consist of Fc-enabled antibodies that bind tumor cells and induce ADCC, and thereby CD16a shedding is a therapeutic target. However, ADAM17 cleaves not only CD16a but also a variety of other proteins that include cytokines, cytokine receptors, and cell adhesion molecules (82). ADAM17 inhibitors can stop the shedding of not only CD16a but also unintended targets, especially if administered *in vivo* given that ADAM17 expression is not restricted to NK cells. To address that limitation, our laboratory developed a novel means to inhibit CD16a shedding, and in a substrate-specific manner that bypasses the broad specificity of ADAM17. We developed the mAb F9H4, which binds CD16a and inhibits the shedding. As a consequence, F9H4 retains CD16a on the cellular surface to enable engagement with the Fc (**Figure 2**). F9H4 is not an agonist or blocker, and was engineered with D265A N297A mutations in the Fc that prevent cell depletion. F9H4 is, therefore, a pharmacologically relevant means of inhibiting CD16a shedding in a highly specific manner that enables physiologically relevant studies. For example, F9H4 not only promoted ADCC *in vitro* against tumor cells that were opsonized with cetuximab, which is an anti-epidermal growth factor receptor antibody, but also synergized with it to inhibit tumor growth in mouse models *in vivo*. Such a biological effect *in vivo* was associated with higher levels of CD16a surface expression in blood NK cells from cetuximab-treated tumor-bearing mice. F9H4 also bound CD16b and inhibited the shedding by neutrophils, and it inhibited CD16a shedding by monocyte-derived macrophages and CD16a/b shedding by tumor-infiltrating leukocytes from patients (72). Therefore, F9H4 selectively inhibits CD16a/b shedding, for being a CD16a/b-targeting mAb, and represents an opportunity to study the physiological significance of the shedding and enhance the efficacy of tumor cell-opsonizing Fc enabled antibodies.

Collectively, the aggregate of data with protease inhibitors, CD16a-S917P, and, recently, with F9H4 consistently show that CD16a shedding inhibits ADCC by preventing receptor engagement.

## The alternative role of CD16a shedding in NK cell-mediated serial killing

NK cells can engage and kill multiple target cells in a subsequent manner, which is called “serial killing.” For example, a single human NK cell can engage and kill between one to five target cells in sixteen hours *in vitro*, and such serial killing can be increased by tumor cell-opsonizing antibodies (83). Antibody afucosylation, which increases the binding affinity to CD16a, further promotes serial killing (84). Strikingly, ADAM17 inhibitors caused the opposite effect, by decreasing the serial killing that occurs through ADCC. ADAM17 inhibitors, which stop CD16a shedding, prevented an NK cell detachment from lymphoma cells that were opsonized with rituximab, and that was followed by an inability of NK cells to move on to the next target cell and NK cell death. Similar results were obtained with an NK cell line that was engineered to express the mutant non-cleavable CD16a (73). Furthermore, a CD30/CD16a-targeting bispecific killer engager (AFM13) required CD16a shedding for optimal induction of serial killing (74). Both rituximab-opsonized lymphoma cells and AFM13-opsonized leukemia cells induced CD16a shedding, which could be stopped by protease inhibitors or genetic engineering of the mutant non-cleavable CD16a (73, 74). In general, protease inhibitors reduced the relative amount of NK cells with more than three contact events for rituximab and more than three killing events for the bi-specific killer engager, respectively (73, 74). Therefore, *in vitro* mechanistic studies revealed that CD16a shedding has a secondary role in ADCC, by promoting the NK cell detachment from target cells for serial killing. While F9H4 promotes ADCC *in vitro* and inhibits tumor growth *in vivo*, it remains to be tested if F9H4 affects serial killing (72).

## CD16a shedding in ADCC: friend or foe?

**Figure 3:**
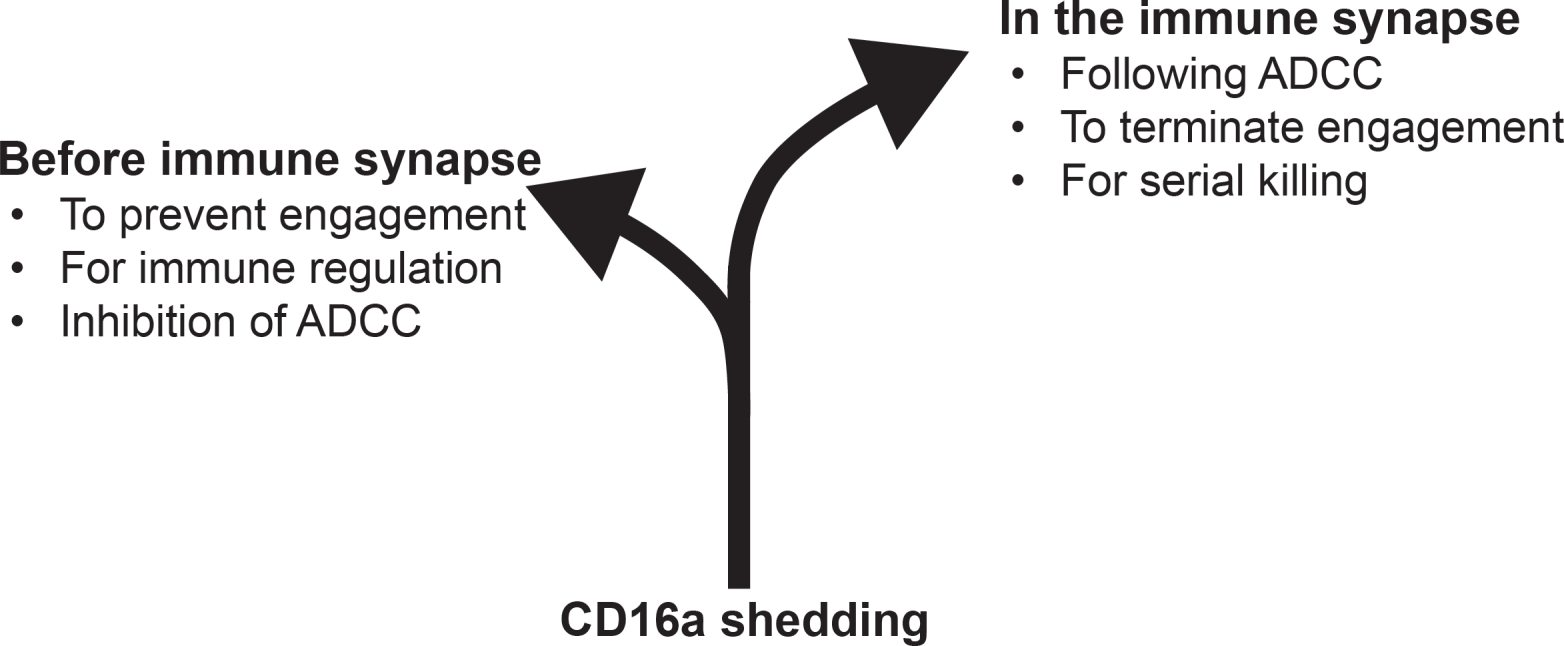
CD16a shedding can be characterized by a bifurcation. Any given CD16a molecule is susceptible to shedding before or after engagement. In the first, before engagement, such a CD16a molecule would be unable to transmit intracellular signaling because it would no longer be on the cellular surface. CD16a shedding before receptor engagement, therefore, inhibits ADCC. On the other hand, in the second scenario, any given CD16a molecule that was not shed before engagement and that bound the Fc can be shed in the immune synapse. In that case, CD16a shedding helps terminate the immune synapse and promote serial killing.

The dual roles of CD16a shedding in negatively regulating ADCC and promoting serial killing are likely mutually exclusive, because any given CD16a molecule is shed either before or after engagement. We, therefore, reason that when CD16a shedding occurs before engagement with the Fc and, thereby, likely before immune synapse formation, it inhibits ADCC, which would not be initiated in the absence of CD16a. Consistent with that notion, NK cells shed CD16a after stimulation with interleukins 12 and 18 or with protein kinase C agonist, which are contexts that do not include immune synapse (63). On the other hand, we also reason that any CD16a molecule that was not cleaved before engagement is susceptible to cleavage in the immune synapse after it is formed, and that may be a mechanism for triggering the disassembling of the immune synapse after cytotoxicity. Consistent with that second notion, CD16a engagement promoted the assembly of the immune synapse, and the inhibition of CD16a cleavage made the immune synapse last longer (73). Another possibility for why CD16a cleavage may be important for terminating the immune synapse is that CD16a physically interacts in *cis* with a cell adhesion molecule, *i.e.*, CD2, which accumulates in the immune synapse (85, 86). By definition, CD16a cannot support cell adhesion without a link with the cytoskeleton, but an in-*cis* interaction between CD16a and CD2 may provide such a link in an indirect manner. It is noticeable that CD2 undergoes shedding in T cells, and thereby one could speculate that a dual shedding of CD16a and CD2 would promote the disingagement (87). Therefore, CD16a shedding appears to have dual roles that are timely exclusive, as it can either occur before or after engagement (**Figure 3**). We reason that CD16a shedding is a therapeutic target in cancer immunology because, when it occurs before engagement, it negatively regulates ADCC, and, thereby, inhibition of CD16a shedding could promote the efficacy of Fc-enabling, tumor cell-opsonizing antibodies. Consistent with that possibility, inhibition of CD16a shedding by protease inhibitors or a CD16a/b-directed antibody and genetically engineered NK cells that expressed the non-cleavable mutant CD16a all enhanced ADCC and promoted the efficacy of several tumor cell-opsonizing antibodies (63, 65–72). For example, we developed this novel mAb (*i.e.*, F9H4) that inhibits CD16a shedding in a substrate-specific manner, which is a unique feature that enabled the demonstration that F9H4 not only promotes ADCC *in vitro* but also inhibits tumor growth *in vivo* (72). Furthermore, other studies demonstrated that tumor-infiltrating NK cells downregulate CD16a (88–91). It remains to be more comprehensively demonstrated how the shedding contributes to CD16a downregulation in the tumor microenvironment, but it is known that the human plasma has soluble CD16a molecules that are shed by NK cells (81). For CD16a shedding to negatively impact serial killing *in vivo*, tumor-infiltrating NK cells must first express CD16a, but it appears that such an expression is relatively low likely due to the shedding that occurs prior to target cell engagement or downregulation at the mRNA level. Therefore, based on the current information from the latest studies, we conclude that CD16a shedding remains as a prime target to promote ADCC by tumor cell-opsonizing antibodies, and that can be hit by protease inhibitors *in vitro*, genetic engineering for cellular therapy, or the CD16a/b-targeted antibody F9H4 that inhibits the shedding in a selective manner that enables *in vivo* studies in immunocompetent hosts.

## The discovery of ectodomain shedding-susceptible proteins may identify novel targets

The number of proteins exceeds substantially the number of genes in any organism, likely through molecular mechanisms such as alternative splicing and post-translational modifications (92). Since ectodomain shedding transforms certain proteins that, in turn, can acquire new functions, it may enable protein diversity that exceeds the number of protein-encoding genes (93). Although a highly informative sheddome database was constructed with publicly available data to include 481 proteins that were experimentally validated for undergoing ectodomain shedding, there is, probably, a myriad of surface proteins that undergo shedding but that were not well characterized yet (94). One recent example is CD200, which is a regulator of inflammatory response. CD200 is a single-pass type-1 transmembrane protein widely expressed and with immune regulatory functions, for example, in the regulation of macrophage lineage (95). CD200 can also be expressed in cancer cells, such as those of neuroblastoma, melanoma, and non-small cell lung cancer (96–98). On the other hand, the receptor for CD200, *i.e.*, CD200R, is an inhibitory receptor, and it is expressed in NK, T, and myeloid cells (99–102). Leukemia cell expression of surface CD200 suppresses NK cell effector functions (103). CD200 also exists in a soluble form that achieves higher concentrations in the plasma of patients with chronic lymphocytic leukemia, and soluble CD200 correlates with worse overall survival of patients with pancreatic cancer (104, 105). Notably, soluble CD200 is generated by tumor cells through ectodomain shedding, and it binds CD200R in NK cells. Given that CD200R is an inhibitory receptor, soluble CD200, by engaging with the receptor, blocked effector functions and induced NK cell apoptosis (106). Therefore, CD200 shedding by tumor cells represents a novel NK cell-targeted mechanism of immune suppression.

## Concluding remarks

Ectodomain shedding is peculiarly relevant in NK cells because they are regulated by the balance between activating and inhibitory receptors, which can be downregulated by shedding upon NK cell stimulation or bind ligands that can be shed by tumor cells as a mechanism of immune evasion. Fc receptors and stress-induced ligands are key examples. We, here, reviewed recent developments in the shedding of CD16a, which is the Fc gamma receptor that triggers ADCC, two types of stress-induced ligands, *i.e.*, MICA/B and B7-H6, and the CD200 inflammatory regulator. The shedding of CD16a and stress ligands determine, at least in part, how many engagement events between receptors and ligands occur upon NK cell encounter with tumor cells, and CD200 shedding enables a contact-independent suppression of NK cells by CD200R, which is an inhibitor receptor. On one hand, ectodomain shedding can interfere with the engagement of activating receptors (*e.g.*, CD16a, NKG2D, and NKp30) to their ligands (the Fc, MICA/B, and B7-H6, respectively) or promote paracrine signaling of an inhibitory receptor (CD200R). On the other hand, the inhibition of shedding has a reverse effect by retaining CD16a on the cell surface for engagement by the Fc, retaining MICA/B and B7-H6 on the surface for NKG2D and NKp30 engagement, respectively, and preventing CD200R engagement by soluble CD200. Therefore, the current but likely challengeable paradigm is that ectodomain shedding inhibits NK cell-driven tumor immunity. Overall, the ectodomain shedding of receptors and ligands represents a research niche with the potential of generating new mechanistic insights into NK cell biology as well as revealing new targets in cancer immunotherapy research. Furthermore, antibodies were specifically designed to inhibit the shedding of CD16a/b and MICA/B (**Table 1**). As those novel approaches for inhibition of ectodomain shedding transition to the clinical trial phase, it is important to consider potential side effects. Stress-induced ligands are selectively expressed in abnormal cells, so there is little possibility of on-target/off-tumor effects. However, MICA/B can be expressed by enterocytes from celiac disease patients in a gluten-based diet (107, 108). MICA/B antibodies, therefore, may cause immune-related adverse events in the gastrointestinal system. Furthermore, inhibition of CD16a shedding may promote side effects commonly observed after certain tumor cell-opsonizing antibodies, such as cetuximab-induced skin rash. NK cell therapies may also have unforeseen distal consequences, due to systemic implications of immune modulation. Therefore, the potential adverse events following the inhibition of ectodomain shedding for NK cell-driven immunotherapy for cancer remain largely speculative, but they should be considered with caution.

**Table 1:** Overview of current ectodomain shedding-targeting therapeutics in cancer immunology.

Target antigens	Antibodies	Mechanism of action	References
MICA/B	7C6	Inhibition of MICA/B shedding, and if engineered as human IgG1 or mouse IgG2a, it engages Fc receptors. 7C6 sequence can also be used as a chimeric antigen receptor for cellular therapy.	(38–40, 42)
MICA/B	CLN-619	A human IgG1 molecule that inhibits MICA/B shedding and engages Fc receptors.	(41)
MICA/B	AHA-1031	Fc-enhanced antibody that inhibits MICA/B shedding and engages with high binding affinity Fc receptors.	(109)
CD16a/b	F9H4	Selective inhibition of CD16a/b shedding and promotion of ADCC.	(72)
CD16a and ADAM17	TAB16	Bispecific engager that binds NK cells through the CD16a-targeting domain and blocks ADAM17.	(110)

List of antibodies that were specifically designed to inhibit the shedding of their target antigens and for use in cancer immunology and immunotherapy.

The molecular mechanisms specifically relevant to the regulation of ectodomain shedding remain largely uncharacterized. For example, although at steady state NK cells express ADAM17, a protease that cleaves CD16a, the shedding is triggered by, for example, cytokine stimulation (63). It is, therefore, possible that ADAM17 is in an inactive state until NK cells are stimulated, and/or NK cell stimulation enables the cleavage by altering CD16a stalk accessibility. On the other hand, stress-induced ligands appear to be constitutively shed by tumor cells (10). Future research directions should focus on the molecular mechanisms that regulate ectodomain shedding to help establish how ectodomain shedding participates in NK cell homeostasis but is dysregulated in pathologies such as cancer.
